# Privacy protection of medical data in social network

**DOI:** 10.1186/s12911-021-01645-0

**Published:** 2021-10-18

**Authors:** Jie Su, Yi Cao, Yuehui Chen, Yahui Liu, Jinming Song

**Affiliations:** 1 School of Information Science and Engineering, University of Jinan, Jinan, 250022 China; 2 Shandong Provincial Key Laboratory of Network Based Intelligent Computing, University of Jinan, Jinan, 250022 China; 3 School of Information Management, Beijing Information Science & Technology University, Beijing, China; 4 Department of Hematopathology and Lab Medicines, H. Lee Moffitt Cancer Center and Research Institute, Tampa, FL 33612 USA

**Keywords:** Medical data, Privacy protection, Cluster, K-anonymity

## Abstract

**Background:**

Protection of privacy data published in the health care field is an important research field. The Health Insurance Portability and Accountability Act (HIPAA) in the USA is the current legislation for privacy protection. However, the Institute of Medicine Committee on Health Research and the Privacy of Health Information recently concluded that HIPAA cannot adequately safeguard the privacy, while at the same time researchers cannot use the medical data for effective researches. Therefore, more effective privacy protection methods are urgently needed to ensure the security of released medical data.

**Methods:**

Privacy protection methods based on clustering are the methods and algorithms to ensure that the published data remains useful and protected. In this paper, we first analyzed the importance of the key attributes of medical data in the social network. According to the attribute function and the main objective of privacy protection, the attribute information was divided into three categories. We then proposed an algorithm based on greedy clustering to group the data points according to the attributes and the connective information of the nodes in the published social network. Finally, we analyzed the loss of information during the procedure of clustering, and evaluated the proposed approach with respect to classification accuracy and information loss rates on a medical dataset.

**Results:**

The associated social network of a medical dataset was analyzed for privacy preservation. We evaluated the values of generalization loss and structure loss for different values of *k* and *a*, i.e. $k$ = {3, 6, 9, 12, 15, 18, 21, 24, 27, 30}, *a* = {0, 0.2, 0.4, 0.6, 0.8, 1}. The experimental results in our proposed approach showed that the generalization loss approached optimal when *a* = 1 and *k* = 21, and structure loss approached optimal when *a* = 0.4 and *k* = 3.

**Conclusion:**

We showed the importance of the attributes and the structure of the released health data in privacy preservation. Our method achieved better results of privacy preservation in social network by optimizing generalization loss and structure loss. The proposed method to evaluate loss obtained a balance between the data availability and the risk of privacy leakage.

## Background

The wide deployment of electronic health record systems has brought convenience to our lives. The need for sharing health data among multiple parties has become evident in several applications, such as decision support, policy development, and data mining [1]. The widespread use of social networks and the integration and fusion of data based on linkage have posed privacy threats to the release of health data and the research of bioinformatics data [2–6]. With the rapid increase in data volume and development of storage cloud platforms, the security of medical data is facing increasing challenges. This is because of the rise of mobile medical industry and the necessary information shared between commercial health insurance information systems, basic medical insurance information systems, and the medical institution information systems. All these contribute to increase in privacy protection difficulties. It is highly likely that patients’ privacy might be disclosed when they use social network tools in daily life or in seeking medical treatment. The disclosure of private information might result in serious consequences to the patients or the society. Therefore, privacy protection is a very important consideration in the field of medical data sharing and distribution.

**Fig. 1:**
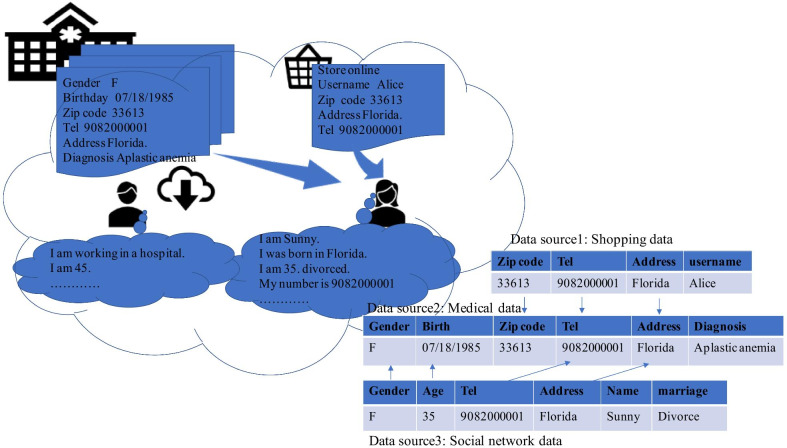
Privacy disclosure caused by social network and by integrating the data source. The data source 1 is the data from the shopping online. The data source 2 is the anonymously published medical data. The attributes of ID, name and marriage have been anonymized. The data source 3 is the data from social network, which also has the attributes of gender, age, phone number and marriage status

According to a survey from a security software company, users in social networks are more likely to encounter the loss of financial information, stolen identity information, and the security threats through software and hardware. In addition, integration and fusion of data based on linkage also results in privacy disclosure, which is demonstrated in Fig. 1. The data source 1 is the data from the shopping online. The data source 2 is the anonymously published medical data. The attributes of ID, name and marriage have been anonymized. The data source 3 is the data from social network, which also has the attributes of gender, age, phone number and marriage status. The attackers can decipher the privacy information (such as the diagnosis), by integrating the data source 1, data source 2, and data source 3.

.

At present, the measures to protect medical data and the privacy of patients mainly include:

- Safely store medical data. File block storage and encryption technology is applied when patients’ files, medical records, and pictures of test results are stored using cloud platforms.
- Enhance the awareness of the protection of patient information. Storage of cards, documents, pictures, or test reports with patients’ information is prohibited. Mention of patient information in public places or unsecured places is not allowed.
- De-identification patient information when possible. Whenever possible, before sharing the necessary medical information, de-identification should be done, especially the patient's name, date of birth, telephone number, address, ID card number, medical record number, photos etc.

Among the above measures, de-identification is very important for privacy protection. Technical efforts are highly encouraged to make published health data both privacy-preserving and useful. The limited release technique selectively publishes data according to specific circumstances by using data generalization and anonymity techniques. For sensitive data, it publishes data with low accuracy or does not publish data. The aim is to find a balance between data availability and privacy protection. It tries to release data with reasonable value, while limits disclosure risk within a reasonable range. These kinds of algorithms have high versatility and wide adaptability. However, data published usually results in a certain degree of loss.

The existing algorithms of privacy assurance are either based on interactive approaches or based on non-interactive approaches. In a non-interactive framework, the owner of the database first anonymizes the raw data and then releases the anonymized version for public usage [1, 7, 8]. Anonymity is the technique to hide or fuzzy the data or the data sources. This kind of technique generally applies some methods to anonymize data by suppression, generalization, analysis, slicing, and separation. Data privacy protection technology in social network is divided into 2 categories: clustering-based method and graph structure modification method. When we use clustering-based method, we divide the nodes and edges of the graph into super nodes and edges, and we hide the sensitive information of nodes and edges in their super classes. Graph structure modification method is similar to K-anonymous, which prevents attackers from using network structure as the background knowledge [9].

The data in social network contains large numbers of sensitive information such as link node attribute, node tag, and graph structure features. Attackers can use either active attack models or passive attack models to dissect and uncover sensitive information. Social network is usually released in the form of a graph. In the graph, each node is described with the entity attribute set. There is a unique identifier for each node. Due to the advantages of the graph, some researchers try to use graph as the tools to study the problem of privacy protection. Some authors [10] categorized the anonymous methods and reviewed anonymous methods on rich graphs. Some other authors [11–15] presented a method of anonymous graph data based on groupings and classing. A clustering approach for data and structural anonymity in social networks was also given [16]. One report [17–19] described how to reserve the privacy of sensitive relationships in graph data. Other reports [20, 21] examined the problem of vertex re-identification from anonymized graphs. Literature [22] proposed methods to release and analyze synthetic graphs in order to protect privacy of individual relationship in the social network. Literature [23] sought a solution to share meaningful graph datasets while preserving privacy. Literatures [24, 25] studied the problem of anonymous graphs in evolving social network. Literatures [26, 27] showed that the true anonymous level of graphs was lower than that obtained by measures such as k-anonymity.

Recent research has indicated that the present models are still vulnerable to various attacks and provide insufficient privacy protection. In this paper, we presented a privacy protection method to release medical data by adopting non-interactive framework [28].

To prevent attacks on network structure, we provided a k-anonymous greedy clustering algorithm based on entities attributes of released social network. In this algorithm, privacy protection algorithm is based on a generalization technique, and a method to evaluate loss was described. It significantly reduces the risk of privacy exposure and at the same time ensures data availability. Moreover, the algorithm is computationally efficient.

## Methods

### The key attributes of medical data in social network

When the medical data is released, each dataset contains a plurality of tuples, and each tuple corresponds to a specific individual member in the society. According to the attribute function and the main objective of privacy protection, the attribute information is divided into three categories. The first category is unique identifier attributes, these attributes can uniquely identify a specific individual member of the community. These include driver license number and social security number (SSN) etc. This kind of attributes are usually hidden before release to the social network. The second category is the approximate identity attributes, which must be presented in a list of published data sheets and external data sources. These include postal codes, home address, etc. The third category is sensitive attributes, which are secret attributes, such as family income or medical history etc. In a social network, the difficulty of privacy protection is increased because the three attributes described above are often interrelated and mutually influenced. In the published shared data table, people often directly remove unique identifies because the unique identifier attributes can clearly identify the individual members of the society with private information. However, the open shared data tables are released with zip codes, gender, birthday and other similar identity. An attacker can often link this data together by the obtained approximate identity attributes and other channels, and can easily identify all the data of the individual members of the community. According to statistics, about 87% of the citizens in the United States can be recognized by means of the approximate identity attributes, such as zip codes, gender, date of birth, etc.

Because of the need for statistics, research, or some other applications, hospitals have to frequently release the patient's data. Table 1 is the patient’s medical information table, in which the sensitive attribute is {disease} and the approximate attributes are {Zip code, age}. Table 2 is the publicly available individual information data table.

**Table 1:** Patient medical information (privacy table)

ID	Zip code	Age	Disease
1	273212	33	heart disease
2	273215	45	heart disease
3	273203	23	influenza
4	273211	29	heart disease
5	273207	50	cancer
6	273206	20	influenza
7	273221	31	A-dis

**Table 2:** Disclosed personal information

ID	Name	Gender	Zip code	Age
1	Mary	Male	273209	29
2	Alice	Female	273212	33
3	David	Male	273211	29
4	Sam	Female	273207	50
5	Joan	Male	273206	20
6	Angle	Female	273221	31

The current practice of preventing the leakage of the patient's privacy information primarily relies on policies and guidelines, such as HIPAA in the USA [29]. However, the reality is that patients’ health records are not perfectly protected while the researchers cannot effectively use them for discoveries. Hospital typically deleted the unique identity of the individual information, and de-identified the unique identity attributes. Although it has protected the individual privacy to certain extent, attackers can still obtain individual privacy information by connecting the approximate identity attributes in Table 1 with the released relevant information in Table 2. For example, if the attacker wants to know Sam’s disease by using the information of his ZIP code and age, it may be inferred that Sam suffered from the disease "cancer". This is a simple link attack. To solve this problem, an attribute information-based clustering algorithm is used in our method.

During the process of social network release, changing the identification information of nodes or changing the structure information by adding or deleting edges is the basic method to protect privacy. Because a large number of historically released data could be collected easily and the information about the nodes can be collected for a certain time period, when the destination node is inserted into the network, attackers sometimes can recognize the target node in the published network. Anonymous methods for such attacks include K degree-anonymity method, K neighborhood anonymous method, and the anonymous method of k sub graph isomorphism [30–32]. However, these three kinds of methods usually result in loss when reconstructing a social network graph.

### K-anonymity based on generalization

K-anonymity is realized by using generalization technology and hiding technology [33]. These two techniques are different from distortion, disturbance, and randomization because they can maintain the authenticity of the data. Attribute-based generalization method can reduce the damage to the original structure and reduce loss.

In order to construct K anonymous, we need to apply generalization techniques not only to the information of nodes, but also to the internal structure of the sub graph and the relationship between sub graphs. The edges used to show the relationship between the sub graphs are used to describe the characteristics of the structure of the network. We construct K-anonymous graph after estimation of the loss, the internal relations of sub graph, and the relationship between sub graphs.

For the graph *G,* there is *G* = (V, E) and *|V|*= *N*, where *N* is the number of the nodes, *V* is the collection of nodes and *E* is the collection of edges. There are the initial partitions for the nodes. Cluster progress needs to fulfill two criterions. The first is that each cluster contains at least k nodes, and the second requirement is to reduce the loss. Therefore, it is necessary to define a method to estimate the loss.

This algorithm clusters *k* nodes to a set with the similar attributes and minimal loss. We record the *V* with an ordered sequence {v_0_, v_1_, ……, v_N_}. The adjacency relationship between nodes is represented by an adjacency matrix A = {a_i,j_}, where *i* = 1, 2, ……, N and *j* = 1, 2, ……, N. When there is direct connection between v_i_ and v_j_, a_i,j_ = 1, otherwise a_i,j_ = 0. The neighborhood can be retrieved. Symmetric binary distance measure was used for this matrix. The node distance and the structure distance are represented by $D\left( {v_{i} ,v_{j} } \right)$ and $D\left( {v_{i} ,s_{k} } \right)$, respectively.

#### Definition 1.

Node distance

$\forall i,j \in 1,2, \ldots ,N,$ the distance between $v_{i}$ and $v_{j}$ is described as:

1 $$D\left( {v_{i} ,v_{j} } \right) = \frac{{\left| d \right|d = {\text{min}}\left( {a_{i,k} + a_{k,q} + \cdots + a_{p,j} } \right)|}}{mn}$$

where $i \ne k \ne q \ne \cdots \ne p \ne j$, $a_{i,k} = a_{k,q} = \cdots = a_{p,j} = 1$, *mn* is the number of nodes in the shortest path.

#### Definition 2.

Structure distance

$\forall i,k \in 1,2, \ldots ,N,$ The distance between $v_{i} (v_{i} \notin s_{k} )$)and $s_{k}$ is described as:

2 $$\forall v_{j} \in s_{k} ,\quad D\left( {v_{i} ,s_{k} } \right) = {{\left( {\mathop \sum \limits_{{v_{j} \in s_{k} }} D\left( {v_{i} ,v_{j} } \right)} \right)} \mathord{\left/ {\vphantom {{\left( {\mathop \sum \limits_{{v_{j} \in s_{k} }} D\left( {v_{i} ,v_{j} } \right)} \right)} {\left| {s_{k} } \right|}}} \right. \kern-\nulldelimiterspace} {\left| {s_{k} } \right|}}$$

where $|s_{k} |$ is the number of nodes in cluster $s_{k}$.

The distance between nodes and the distance between a node and a cluster are in the interval of [0, 1]. For graph G, the node with the maximum degree is selected to be the center of a new cluster. Unallocated nodes with the minimize distance to the structure was selected to form a new cluster.

### Loss evaluation

According to the attributes of the nodes, the loss of cluster includes generalization loss and structure loss [29]. Generalization loss is used to calculate the loss of the descriptive information for the node [32], which is defined as:

3 $${\text{GLoss}}\left( {{\text{G}},PS} \right) = \frac{{\mathop \sum \nolimits_{{{\text{j}} = 1}}^{{\text{m}}} \left( {\left| {{\text{s}}_{{\text{j}}} } \right|} \right) \cdot \left( {{\text{Attr}}\left( {{\text{s}}_{{\text{j}}} ,{\text{N}}} \right) + {\text{Cate}}\left( {{\text{s}}_{{\text{j}}} ,{\text{C}}} \right)} \right)}}{{{\text{n}} \cdot \left( {{\text{p}} + {\text{q}}} \right)}}$$

where $P{\text{S}} = \left\{ {{\text{s}}_{1} ,{\text{s}}_{2} , \ldots ,{\text{s}}_{{\text{m}}} } \right\}$ is the partition, $\left| {{\text{s}}_{{\text{j}}} } \right|$ is the cardinality of cluster ${\text{s}}_{{\text{j}}} ,$ ${\text{N}} = \{ {\text{N}}_{1} ,{\text{N}}_{2} , \ldots ,{\text{N}}_{{\text{p}}} \}$ is the set of numerical attributes and ${\text{C}} = \{ {\text{C}}_{1} ,{\text{C}}_{2} , \ldots ,{\text{C}}_{{\text{q}}} \}$ is the set of categorical attributes. ${\text{Attr}}\left( {{\text{s}}_{{\text{j}}} ,{\text{N}}} \right)$ and ${\text{Cate}}\left( {{\text{s}}_{{\text{j}}} ,{\text{C}}} \right)$ are the generalization loss factors caused by generalizing attributes, which are defined as:

4 $${\text{Attr}}\left( {{\text{s}}_{{\text{j}}} ,{\text{N}}} \right) = \mathop \sum \limits_{{{\text{k}} = 1}}^{{\text{p}}} \frac{{{\text{size}}\left( {{\text{gen}}\left( {{\text{s}}_{{\text{j}}} } \right)\left[ {{\text{N}}_{{\text{k}}} } \right]} \right)}}{{\left( {\max_{{{\text{X}} \in {\text{N}}}} \left( {{\text{X}}\left[ {{\text{N}}_{{\text{k}}} } \right]} \right) - \min_{{{\text{X}} \in {\text{N}}}} \left( {{\text{X}}\left[ {{\text{N}}_{{\text{k}}} } \right]} \right)} \right)}}$$

5 $${\text{Cate}}\left( {{\text{s}}_{{\text{j}}} ,{\text{C}}} \right) = \mathop \sum \limits_{{{\text{k}} = 1}}^{{\text{q}}} \frac{{{\text{height}}\left( {{\text{M}}\left( {{\text{gen}}\left( {{\text{s}}_{{\text{j}}} } \right)\left[ {{\text{C}}_{{\text{k}}} } \right]} \right)} \right)}}{{{\text{height}}\left( {{\text{H}}_{{{\text{C}}_{{\text{k}}} }} } \right)}}$$

where ${\text{gen}}\left( {{\text{s}}_{{\text{j}}} } \right)$ is the generalization information of cluster ${\text{s}}_{{\text{j}}}$, and it has the values of attribute, numerical or categorical, the most specific common generalized value for all the values of attributes from ${\text{s}}_{{\text{j}}}$ sets. ${\text{gen}}\left( {{\text{s}}_{{\text{j}}} } \right)\left[ {{\text{N}}_{{\text{k}}} } \right]$ is the interval between $\left[ {\min \left\{ {{\text{X}}^{1} \left[ {{\text{N}}_{{\text{k}}} } \right], \ldots ,{\text{X}}^{{\text{u}}} \left[ {{\text{N}}_{{\text{k}}} } \right]} \right\},{\text{max}}\left\{ {{\text{X}}^{1} \left[ {{\text{N}}_{{\text{k}}} } \right], \ldots ,{\text{X}}^{{\text{u}}} \left[ {{\text{N}}_{{\text{k}}} } \right]} \right\}} \right]$. ${\text{size}}\left( {{\text{gen}}\left( {{\text{s}}_{{\text{j}}} } \right)\left[ {{\text{N}}_{{\text{k}}} } \right]} \right)$ is shown as formula:

6 $${\text{size}}\left( {{\text{gen}}\left( {{\text{s}}_{{\text{j}}} } \right)\left[ {{\text{N}}_{{\text{k}}} } \right]} \right) = \max \left\{ {{\text{X}}^{1} \left[ {{\text{N}}_{{\text{k}}} } \right], \ldots ,{\text{X}}^{{\text{u}}} \left[ {{\text{N}}_{{\text{k}}} } \right]} \right\} - \min \left\{ {{\text{X}}^{1} \left[ {{\text{N}}_{{\text{k}}} } \right], \ldots ,{\text{X}}^{{\text{u}}} \left[ {{\text{N}}_{{\text{k}}} } \right]} \right\}$$

The hierarchy attribute associated with the classification is defined as ${\text{H}}_{{{\text{C}}_{{\text{k}}} }}$. ${\text{gen}}\left( {{\text{s}}_{{\text{j}}} } \right)\left[ {{\text{C}}_{{\text{k}}} } \right]$ is defined as the recent ancestors. ${\text{M}}\left( {{\text{gen}}\left( {{\text{s}}_{{\text{j}}} } \right)\left[ {{\text{C}}_{{\text{k}}} } \right]} \right)$ is ${\text{H}}_{{{\text{C}}_{{\text{k}}} }}$ when ${\text{gen}}\left( {{\text{s}}_{{\text{j}}} } \right)\left[ {{\text{C}}_{{\text{k}}} } \right]$ is the root of the sublayers. height (${\text{H}}_{{{\text{C}}_{{\text{k}}} }}$) is defined as the height of sub layer.

Parameter $\alpha$ and $\beta$ are set by the user and are used to control the relative information importance of the nodes and the structure.

The other loss is structure loss, which occurs when masking the graph G based on partition *PS* $= \left\{ {{\text{s}}_{1} ,{\text{s}}_{2} , \ldots ,{\text{s}}_{{\text{m}}} } \right\}$. The structural information includes all inter-cluster information and intra-cluster structural information. ${\text{SLoss}}\left( {{\text{G}},PS} \right)$ is defined in [34], which is shown as formula:

7 $${\text{SLoss}}\left( {{\text{G}},PS} \right) = \frac{{\mathop \sum \nolimits_{{{\text{j}} = 1}}^{{\text{m}}} \left( {{\text{intraSL}}\left( {{\text{s}}_{{\text{j}}} } \right)} \right) + \mathop \sum \nolimits_{{{\text{i}} = 1}}^{{\text{m}}} \mathop \sum \nolimits_{{{\text{j}} = {\text{i}} + 1}}^{{\text{m}}} \left( {{\text{interSL}}\left( {{\text{s}}_{{\text{i}}} ,{\text{s}}_{{\text{j}}} } \right)} \right)}}{{\left( {{\text{n}} \cdot \left( {{\text{n}} - 1} \right)/4} \right)}}$$

where $\mathop \sum \nolimits_{{{\text{j}} = 1}}^{{\text{m}}} \left( {{\text{intraSL}}\left( {{\text{s}}_{{\text{j}}} } \right)} \right)$ is the intra-cluster structure loss and $\mathop \sum \nolimits_{{{\text{i}} = 1}}^{{\text{m}}} \mathop \sum \nolimits_{{{\text{j}} = {\text{i}} + 1}}^{{\text{m}}} \left( {{\text{interSL}}\left( {{\text{s}}_{{\text{i}}} ,{\text{s}}_{{\text{j}}} } \right)} \right)$ is the inter-cluster structure loss, satisfying factors:

8 $${\text{intraSL}}\left( {{\text{s}}_{{\text{j}}} } \right) = 2 \cdot \left| {{\text{E}}_{{{\text{s}}_{{\text{j}}} }} } \right| \cdot \left( {1 - {\raise0.7ex\hbox{${\left| {{\text{E}}_{{{\text{s}}_{{\text{j}}} }} } \right|}$} \!\mathord{\left/ {\vphantom {{\left| {{\text{E}}_{{{\text{s}}_{{\text{j}}} }} } \right|} {\left( {\begin{array}{*{20}c} {\left| {{\text{s}}_{{\text{j}}} } \right|} \\ 2 \\ \end{array} } \right)}}}\right.\kern-\nulldelimiterspace} \!\lower0.7ex\hbox{${\left( {\begin{array}{*{20}c} {\left| {{\text{s}}_{{\text{j}}} } \right|} \\ 2 \\ \end{array} } \right)}$}}} \right)$$

9 $${\text{interSL}}\left( {{\text{s}}_{{\text{i}}} ,{\text{s}}_{{\text{j}}} } \right) = 2 \cdot \left| {{\text{E}}_{{{\text{s}}_{{\text{i}}} ,{\text{s}}_{{\text{j}}} }} } \right| \cdot \left( {1 - {\raise0.7ex\hbox{${\left| {{\text{E}}_{{{\text{s}}_{{\text{i}}} ,{\text{s}}_{{\text{j}}} }} } \right|}$} \!\mathord{\left/ {\vphantom {{\left| {{\text{E}}_{{{\text{s}}_{{\text{i}}} ,{\text{s}}_{{\text{j}}} }} } \right|} {\left( {\left| {{\text{s}}_{{\text{i}}} } \right| \cdot \left| {{\text{s}}_{{\text{j}}} } \right|} \right)}}}\right.\kern-\nulldelimiterspace} \!\lower0.7ex\hbox{${\left( {\left| {{\text{s}}_{{\text{i}}} } \right| \cdot \left| {{\text{s}}_{{\text{j}}} } \right|} \right)}$}}} \right)$$

When $\left| {{\text{E}}_{{{\text{s}}_{{\text{i}}} ,{\text{s}}_{{\text{j}}} }} } \right| = \frac{{\left( {\left| {{\text{s}}_{{\text{i}}} } \right| \cdot \left| {{\text{s}}_{{\text{j}}} } \right|} \right)}}{2}$, structure loss achieves the maximum value. The maximum loss and anonymous graph construction process in the class structure is defined as the maximum loss:

10 $$\max \mathop \sum \limits_{{{\text{j}} = 1}}^{{\text{m}}} {\text{intraSL}}\left( {{\text{s}}_{{\text{j}}} } \right) = \mathop \sum \limits_{{{\text{j}} = 1}}^{{\text{m}}} \left( {\frac{{\left| {{\text{s}}_{{\text{j}}} } \right| \cdot \left( {\left| {{\text{s}}_{{\text{j}}} } \right| - 1} \right)}}{4}} \right) = \frac{1}{4}\mathop \sum \limits_{{{\text{j}} = 1}}^{{\text{m}}} \left| {{\text{s}}_{{\text{j}}} } \right|^{2} - \frac{1}{4}\left( {\mathop \sum \limits_{{{\text{j}} = 1}}^{{\text{m}}} \left| {{\text{s}}_{{\text{j}}} } \right|} \right)$$

11 $$\max \left( {\mathop \sum \limits_{{{\text{i}} = 1}}^{{\text{m}}} \mathop \sum \limits_{{{\text{j}} = {\text{i}} + 1}}^{{\text{m}}} \left( {{\text{interSL}}\left( {{\text{s}}_{{\text{j}}} ,{\text{s}}_{{\text{j}}} } \right)} \right)} \right) = \mathop \sum \limits_{{{\text{i}} = 1}}^{{\text{m}}} \mathop \sum \limits_{{{\text{j}} = {\text{i}} + 1}}^{{\text{m}}} \left( {\frac{{\left| {{\text{s}}_{{\text{i}}} } \right| \cdot \left| {{\text{s}}_{{\text{j}}} } \right|}}{4}} \right)$$

where ${\text{SLoss}}\left( {{\text{G}},PS} \right)$ is a value in interval [0, 1].

For an initial social network G, we can obtain a partition $PS = \left\{ {{\text{s}}_{1} ,{\text{s}}_{2} , \ldots ,{\text{s}}_{{\text{m}}} } \right\}$ using the graph anonymous cluster algorithm. $\left\{ {{\text{SC}}_{1} ,{\text{SC}}_{2} , \ldots ,{\text{SC}}_{{\text{m}}} } \right\}$ is the focus node set corresponding to the cluster set $\left\{ {{\text{s}}_{1} ,{\text{s}}_{2} , \ldots ,{\text{s}}_{{\text{m}}} } \right\}$. $s_{{\text{i}}} = \left[ {{\text{gen}}\left( {{\text{s}}_{{\text{i}}} } \right),\left( {\left| {{\text{s}}_{{\text{i}}} } \right|,\left| {{\text{E}}_{{{\text{s}}_{{\text{i}}} }} } \right|} \right)} \right],{ }$ where $\left( {\left| {{\text{s}}_{{\text{i}}} } \right|,\left| {{\text{E}}_{{{\text{s}}_{{\text{i}}} }} } \right|} \right)$ is the intra-cluster generalization pair, $s_{{\text{i}}} \cap s_{{\text{j}}} = \emptyset$, ${\text{i}},{\text{j}} = 1\ldots {\text{m}},\; {\text{and}}\;{\text{i}} \ne {\text{j}}.$ The masked social network is defined as:

12 $${\text{G}}_{{\text{m}}} = \left( {\left\{ {{\text{s}}_{1} ,{\text{s}}_{2} , \ldots ,{\text{s}}_{{\text{m}}} } \right\},{ }\left\{ {{\text{s}}_{1} ,{\text{s}}_{2} , \ldots ,{\text{s}}_{{\text{m}}} } \right\} \times \left\{ {{\text{s}}_{1} ,{\text{s}}_{2} , \ldots ,{\text{s}}_{{\text{m}}} } \right\}} \right)$$

In the above definition, for $\forall {\text{ e }}\left( {{\text{v}}_{{\text{k}}} ,{\text{v}}_{{\text{p}}} } \right),$ there is an edge $\left( {{\text{s}}_{{\text{i}}} ,{\text{s}}_{{\text{j}}} } \right) \in \left\{ {{\text{s}}_{1} ,{\text{s}}_{2} , \ldots ,{\text{s}}_{{\text{m}}} } \right\} \times \left\{ {{\text{s}}_{1} ,{\text{s}}_{2} , \ldots ,{\text{s}}_{{\text{m}}} } \right\}$, ${\text{where v}}_{{\text{k}}} \in {\text{s}}_{{\text{i}}} {\text{ and v}}_{{\text{p}}} \in {\text{s}}_{{\text{j}}} { }$.

The anonymized graph was created by using generalization information and edge intra-cluster generation with a cluster and edge inter-cluster generalization between any two clusters. All nodes from the cluster ${\mathrm{s}}_{1}$ collapsed into the generalized node ${\mathrm{SC}}_{1}$. These nodes are indistinguishable from each other. If the condition ${|\mathrm{s}}_{1}|\ge \mathrm{k}$ is met, a k-anonymous social network can be constructed. When the social network is evolving, we first evaluate the change of structure in the published social network.

A k-anonymous greedy clustering algorithm based on entities attributes of released social network is shown as the following:
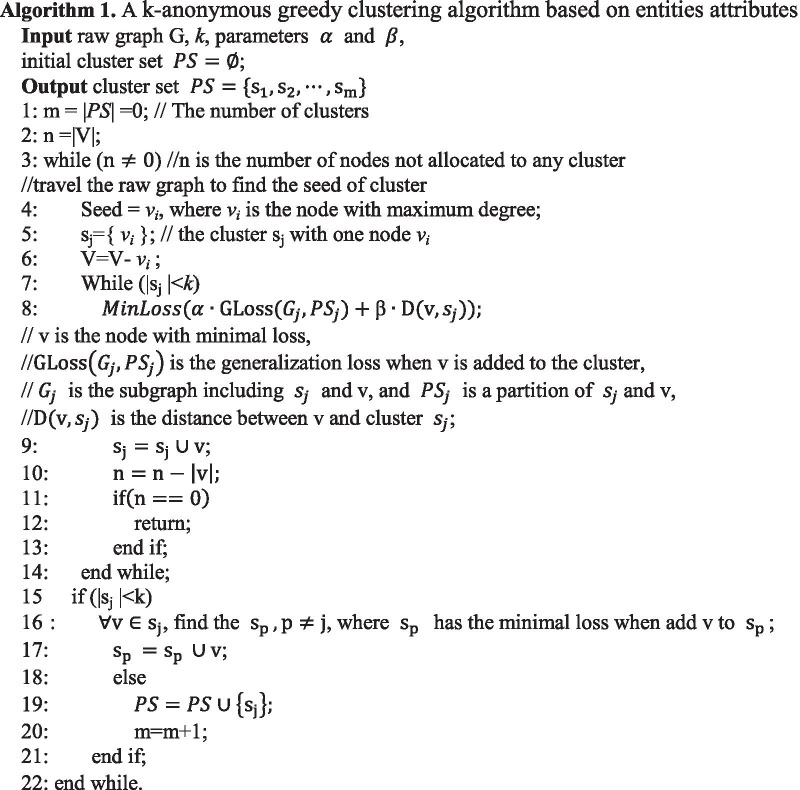


## Results

### Simulation experiments

Our method was tested on a social network associated with a medical dataset. Table 3 shows the basic medical records of 60 patients.

**Table 3:** Medical data of 60 patients

Record No	Gender	Age	Zip code	Marriage	Smoke	Diagnosis
1	F	56	33613	Divorce	Y	AA
2	F	60	33647	Marriage	N	Diabetes
3	M	81	34660	Single	N	AA
4	M	44	32801	Divorce	Y	AA
5	M	56	32211	Marriage	N	AA
6	M	34	32868	Marriage	N	Normal
7	F	73	34768	Single	N	AA
8	M	77	33102	Marriage	N	AA
9	F	84	32855	Single	N	AA
10	F	68	33709	Marriage	N	ACML
11	F	66	34302	Marriage	Y	Diabetes
12	M	53	34565	Single	Y	AML
13	M	59	32652	Marriage	Y	AITCL
14	F	63	33615	Marriage	N	Normal
15	F	19	75865	Single	N	AML
16	M	38	33650	Single	Y	Normal
17	M	56	75677	Marriage	N	Normal
18	F	67	33218	Marriage	N	AA
19	M	65	34813	Marriage	N	ACML
20	M	71	32556	Marriage	N	Normal
21	F	67	33451	Marriage	Y	AML FROM MDS
22	M	60	33648	Marriage	Y	Normal
23	F	56	33613	Divorce	N	AA
24	F	60	33647	Marriage	Y	AA
25	M	51	34660	Single	N	ACML
26	M	34	32801	Divorce	N	Normal
27	M	56	32211	Marriage	N	AML FROM MDS
28	M	34	32868	Marriage	Y	ACML
29	M	38	72868	Marriage	N	ACML
30	F	73	34768	Single	N	AA
31	F	57	33102	Marriage	N	AA
32	F	84	32855	Single	N	AA
33	F	60	33709	Marriage	N	Diabetes
34	F	66	34302	Marriage	Y	ACML
35	M	73	34565	Single	N	AML
36	M	59	32652	Marriage	N	AITCL
37	F	43	33615	Marriage	Y	AML
38	F	29	75865	Single	Y	AML
39	M	48	33650	Single	N	AML
40	M	76	75677	Marriage	N	Normal
41	F	37	33218	Marriage	Y	AML
42	M	45	34813	Marriage	N	AML
43	M	51	32556	Marriage	N	AITCL
44	F	67	33451	Marriage	N	AML
45	M	60	33648	Marriage	N	Normal
46	F	56	33613	Divorce	Y	AA
47	F	63	33647	Marriage	N	AA
48	M	56	34660	Marriage	N	Normal
49	M	34	32801	Divorce	Y	AML
50	M	52	32211	Marriage	N	AITCL
51	M	34	32868	Marriage	N	Normal
52	F	43	34768	Single	Y	AA
53	M	38	75685	Marriage	Y	AML FROM MDS
54	M	42	72512	Marriage	Y	ACML
55	M	26	75828	Single	Y	ACML
56	F	33	34574	Single	N	AA
57	M	47	34543	Marriage	Y	ACML
58	F	62	32767	Marriage	Y	Normal
59	M	55	75926	Marriage	Y	AML
60	M	42	75384	Marriage	Y	AML

**Fig. 2:**
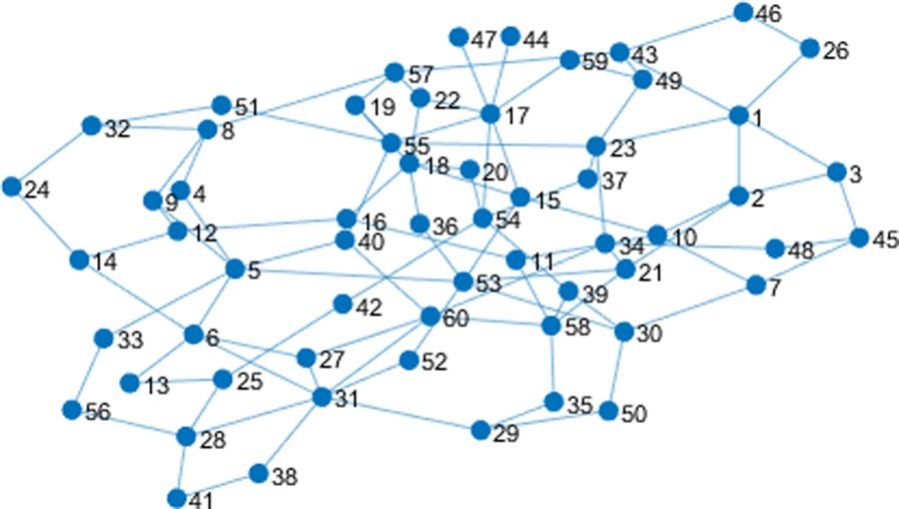
The social network associated with the medical dataset. 60 patients from Table 3 are 60 entities in the social network. Some quasi-identifiers, such as the age, gender, zip codes, and marriage status can be retrieved in this social network

Unique identifier such as driver license or SSN has been removed. There are still some quasi-identifiers, such as the age, gender, zip codes, and marriage status. The relation network corresponding to the entities in Table 3 is shown as Fig. 2.

**Fig. 3:**
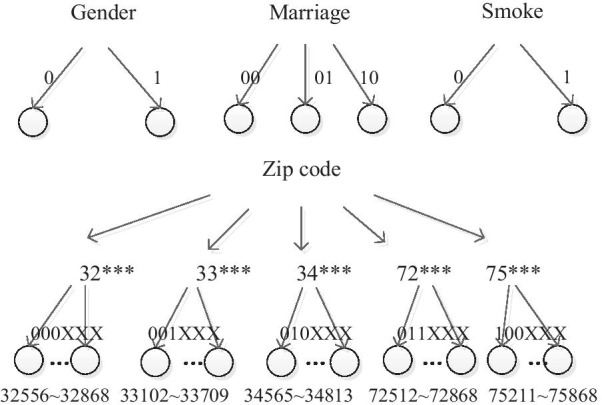
Hierarchy structures of the categorical attributes. Attributes of 60 entities include numerical attributes, i.e. Age, and categorical attributes, i.e. Gender, Marriage, Smoke, Zip code. Categorical attributes are expressed as hierarchy structures

There are 60 nodes (entities) in this social network. When two entities have some relationship, we link them with one edge. We used our anonymous method to protect the privacy of patients. Attribute set of each node can be denoted as $Attr=\mathrm{N}\cup \mathrm{C}$. The set of numerical attributes is defined as $\mathrm{N}=\{\mathrm{Age}\}$. The set of categorical attributes is defined as $\mathrm{C}=\{\mathrm{ Gender},\mathrm{ Marriage},\mathrm{Smoke},\mathrm{Zip code}\}$. The hierarchical structures of the categorical attributes are shown in Fig. 3.

**Fig. 4:**
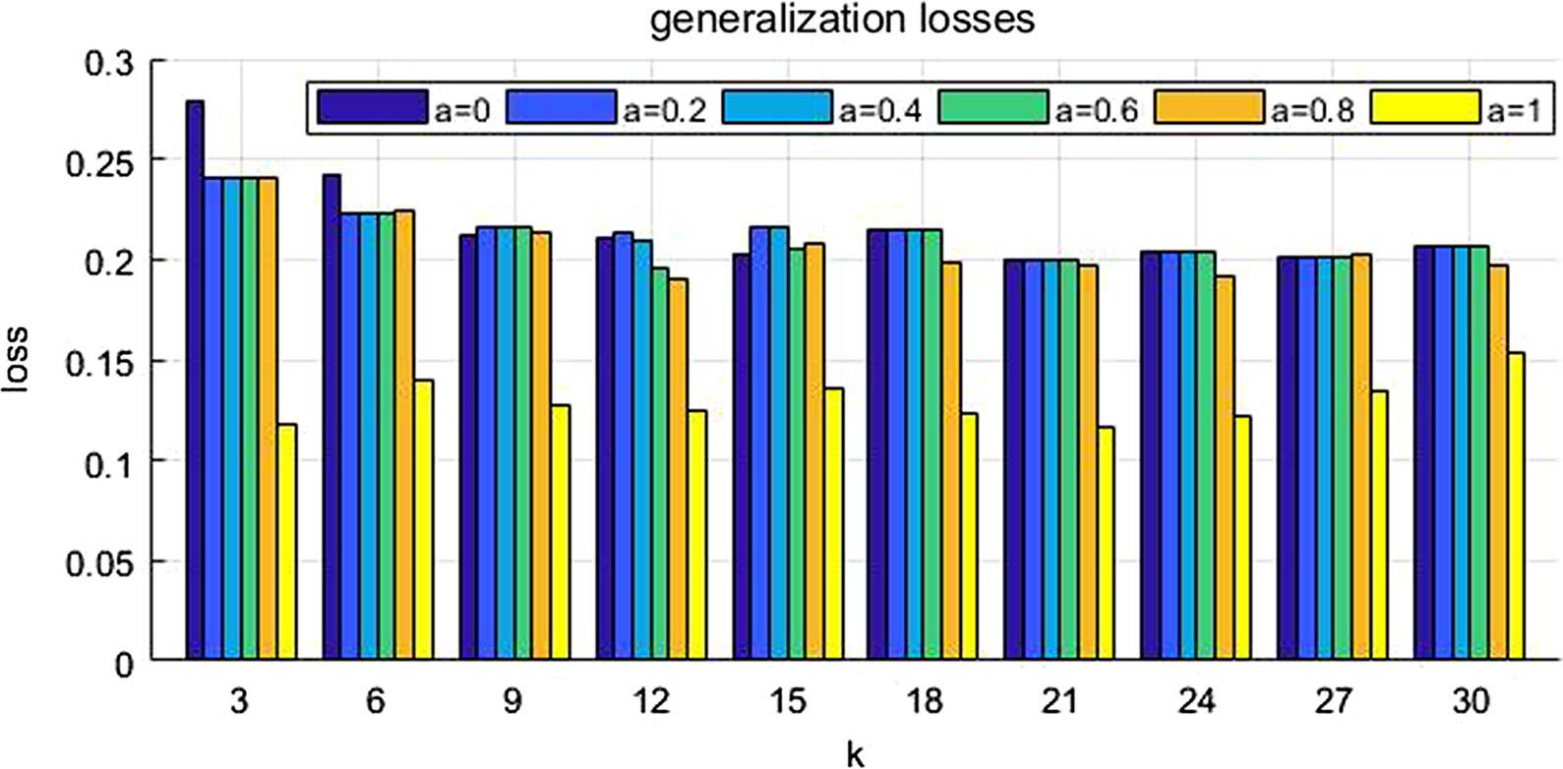
Generalization losses. Generalization losses are tested during anonymity clustering for different values of the parameter *k*, i.e.* k* = {3, 6, 9, 12, 15, 18, 21, 24, 27, 30}

**Fig. 5:**
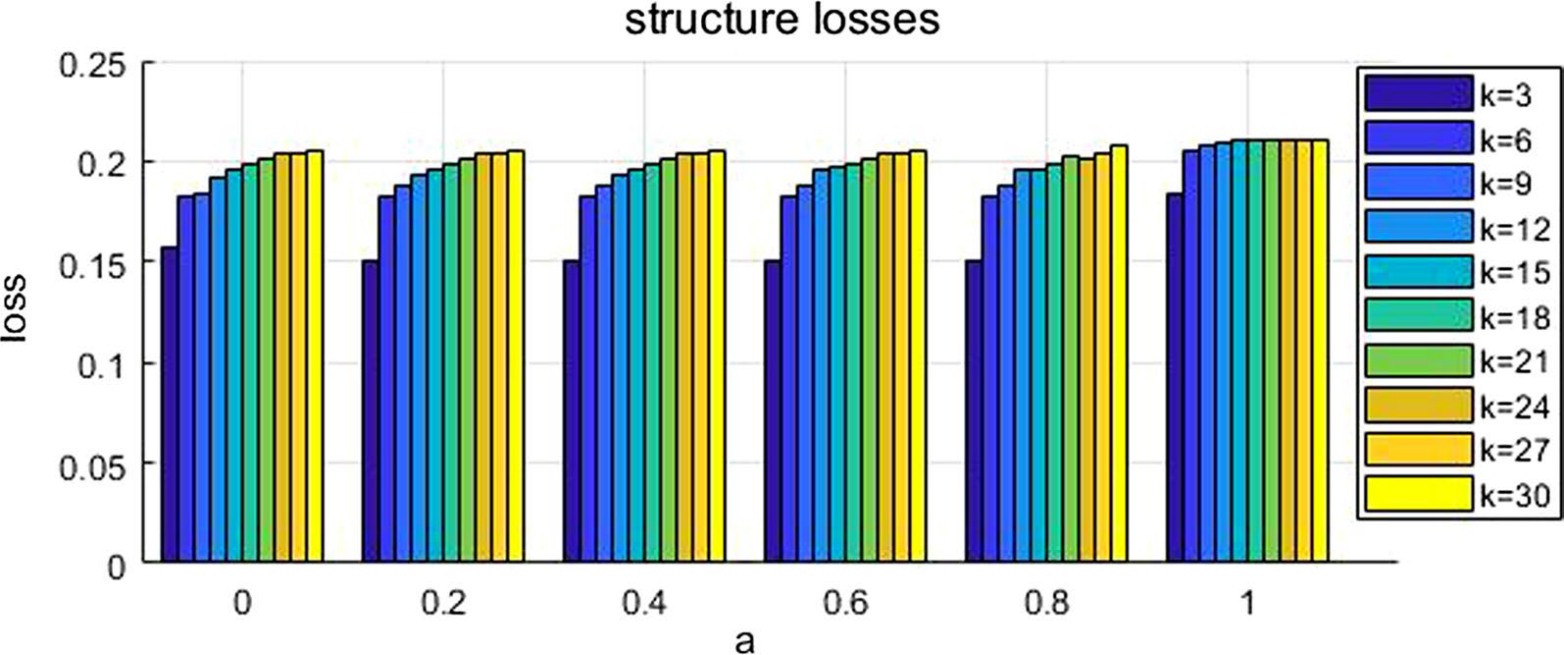
Structure losses. Structure losses are tested during anonymity clustering for different values of the parameter *a*, i.e. *a* = {0, 0.2, 0.4, 0.6, 0.8, 1}

We tested the generalization losses and the structure losses during anonymity clustering for different values of the parameters *k* and *a*, *i.e. k* = {3, 6, 9, 12, 15, 18, 21, 24, 27, 30}, *a* = {0, 0.2, 0.4, 0.6, 0.8, 1}. Figure 4 shows the generalization losses. Figure 5 shows the structure losses for the anonymous cluster. When parameter k is fixed, generalization loss tends to be less when parameter *a* becomes bigger. When parameter *a* is fixed, structure loss tends to be more when k becomes bigger.

Tables 4 and 5 show the generalization losses and the structure losses separately when k and a take different value. The generalization loss approached optimal when *k* = 21 and *a* = 1, and structure loss approached optimal when *k* = 3 and *a* = 0.2, 0.4, 0.6, 0.8. It can be seen that the value of *k* mostly affects the structural losses and the value of *a* mostly affects the generalization losses.

**Table 4:** Generalization losses. The generalization loss approached optimal and it is 0.116333 when k = 21 and a = 1

*k*	*a*					
	0	0.2	0.4	0.6	0.8	1
3	0.278462	0.240051	0.240051	0.240051	0.240051	0.118513
6	0.242615	0.22241	0.22241	0.22241	0.224	0.140103
9	0.211692	0.216103	0.216103	0.216103	0.213051	0.127205
12	0.211026	0.213744	0.209615	0.195641	0.190718	0.124282
15	0.202949	0.215513	0.215513	0.204872	0.207949	0.135513
18	0.214872	0.214872	0.214872	0.214872	0.198615	0.123538
21	0.200333	0.200333	0.200333	0.200333	0.196923	**0.116333**
24	0.203256	0.203256	0.203256	0.203256	0.192	0.12141
27	0.201795	0.201795	0.201795	0.201795	0.202949	0.134615
30	0.206667	0.206667	0.206667	0.206667	0.197308	0.152821

**Table 5:** Structure losses. The structural loss approached optimal and it is 0.150157 when k = 3 and a = 0.2, 0.4, 0.6, 0.8. The structural loss approached optimal and it is 0.150157 when k = 3 and *a* = 0.2, 0.4, 0.6, 0.8

*k*	*a*					
	0	0.2	0.4	0.6	0.8	1
3	0.156685	**0.150157**	**0.150157**	**0.150157**	**0.150157**	0.184306
6	0.182498	0.182172	0.182172	0.182172	0.182097	0.205461
9	0.18415	0.188338	0.188338	0.188338	0.188031	0.208391
12	0.192364	0.19258	0.192517	0.195209	0.196268	0.209272
15	0.195478	0.195952	0.195952	0.197481	0.195349	0.210337
18	0.198426	0.198426	0.198426	0.198426	0.198616	0.210719
21	0.200807	0.200807	0.200807	0.200807	0.201999	0.210436
24	0.204509	0.204509	0.204509	0.204509	0.201373	0.210497
27	0.204427	0.204427	0.204427	0.204427	0.204442	0.211029
30	0.205762	0.205762	0.205762	0.205762	0.207728	0.211137

**Fig. 6:**
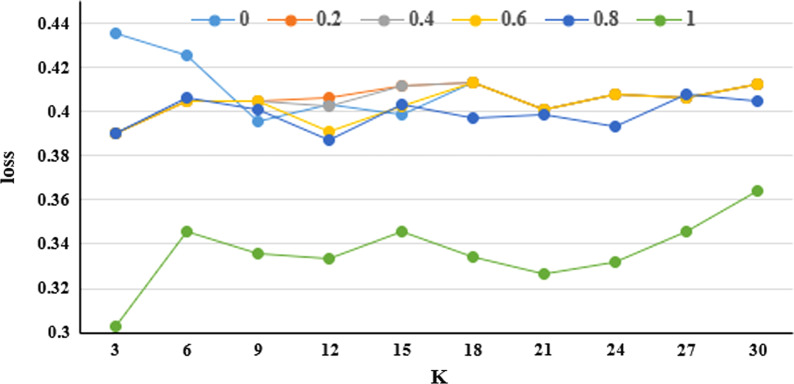
The final losses of the clusters. The final losses of the clusters include structure losses and generalization losses

The final losses of the clusters, which include structure losses and generalization losses, are shown in Fig. 6. We can see that the losses are relatively stable when *a* = 0.2, 0.4, 0.6 0.8.

**Fig. 7:**
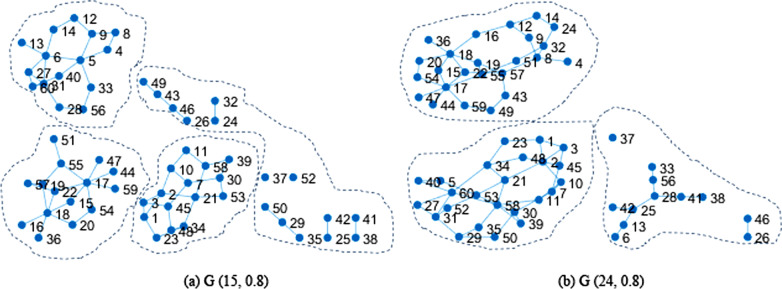
Clustering results. **a** Is the result of clustering when *k* = 15 and *a* = 0.8. **b** Is the result of clustering when* k* = 24 and *a* = 0.8

Figure 7 shows the clustering results based on loss estimation. (a) is the result of clustering when *k* = 15 and *a* = 0.8. (b) is the result of clustering when* k* = 24 and *a* = 0.8. We can see that the clustering results is dependent on the value of *k.* The entities with similar attributes and shortest distance in the network tend to be in the same cluster through anonymous clustering. This method helps to control the scope of information dissemination.

**Fig. 8:**
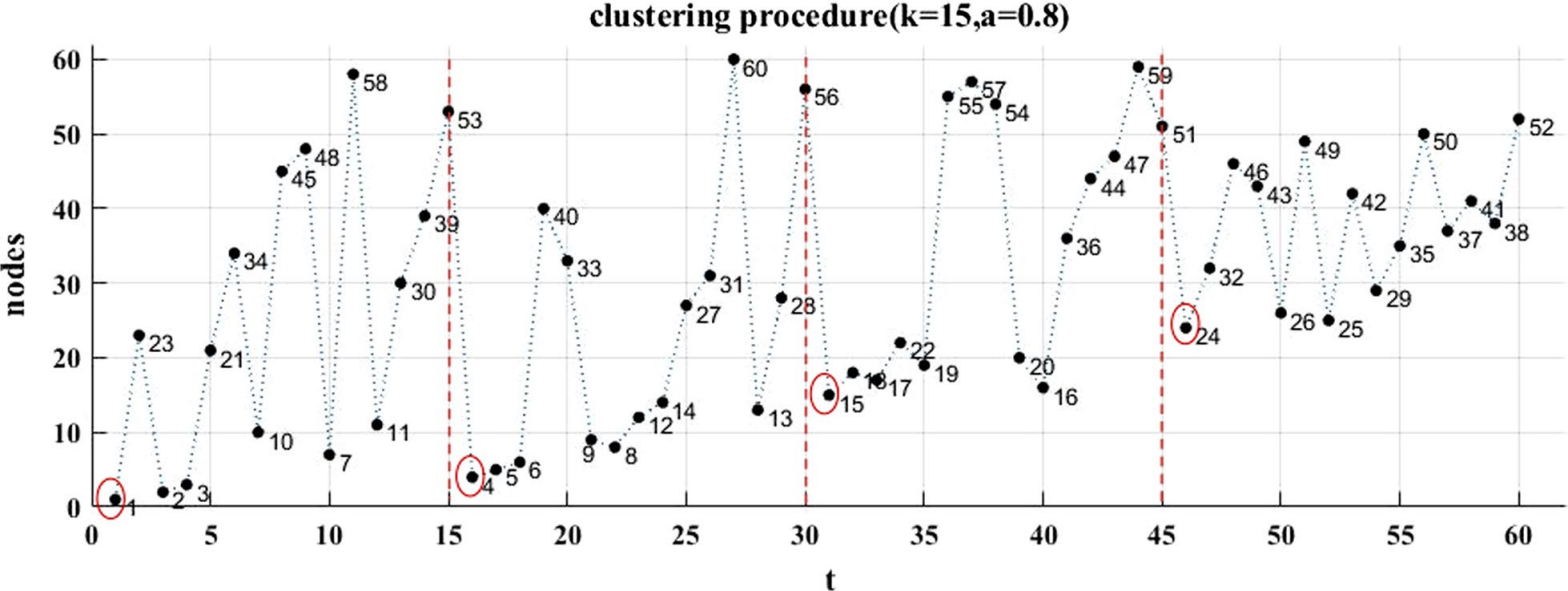
Clustering procedure. The entities in red circles are the centers of clusters

Figure 8 shows a clustering procedure when *k* = 15 and *a* = 0.8, which correspond to those in Fig. 7a. It is easier to locate the center of each cluster and to distinguish the entities from each cluster through this visual display.

## Discussion

Medical researches require the collection of a large number of medical data for experiments and analysis. However, medical data is highly sensitive, and patients' privacy needs to be protected. Leakage of sensitive information is becoming a more and more problem due to increased information exchange in social networks. In order to protect the privacy of medical data to the greatest extent, this paper proposed a privacy protection method based on social network structure and key attributes of network entities. This method helps to control the exposure of sensitive information in social network by the clustering method.

Although unique identifiers might have been removed in medical data, some quasi-identifiers, such as the age, the gender, the zip codes, and the marriage status, which are often used in medicine researches, can still be queried to identify the patients to some extent. In this paper, we divided the key attributes into two categories, numerical attributes and categorical attributes. Categorical attributes are assigned to hierarchical structures, which are shown in Fig. 3. The distance between entities is also used in clustering algorithm. This distance is not only associated to the hierarchical distance of entities in the structure, but also associated to the numerical space distance of entity attributes. We utilized a structure loss and a generalization loss to evaluate the clustering algorithm, and the results are shown in Tables 4 and 5. In our experiments on a medical social data network with 60 entities, the minimum clustering loss is 0.302819, which is shown in Fig. 6. A cluster visualization demonstration (in Fig. 8) displays the center of each cluster and the entities in each cluster.

## Conclusion

In this paper, we studied the privacy protection of medical data in social network. We used medical data sharing as an example to discuss the importance of the attributes in the privacy protection of health data. Nodes (entities) were clustered according to the features of attribute values and the distance of nodes in the network. The entities with similar attributes and shortest distance in the network tends to be in the same cluster through anonymous clustering. This method helps to control the scope of information dissemination. In some sub-network controlled by clusters, the sensitive data will be published with low accuracy or will not be published. The method can be used for real-time analysis.

Since the anonymous clustering in the network usually results in loss, this paper also paid special attention to the estimation algorithm for loss. A K-anonymous method based on attributes and distance clustering was proposed to estimate the loss during clustering. It tries to release data with reasonable value, while controlling disclosure risk within a reasonable range. The aim is to find a balance between data availability and privacy protection. The experiments on a social network associated with a medical dataset demonstrated our clustering procedure and the clustering results, and the usefulness of our method to protect privacy by controlling information release.

## Data Availability

The codes and the data of this manuscript are available at: https://github.com/SuJie-Med/Medical-data.

## Abbreviations

SSN: Social Security Number
HIPAA: Health Insurance Portability and Accountability Act

## About this supplement

This article has been published as part of BMC Medical Informatics and Decision Making Volume 21 Supplement 1, 2021: Proceedings of the 2019 International Conference on Intelligent Computing (ICIC 2019): medical informatics and decision making. The full contents of the supplement are available online at https://bmcmedinformdecismak.biomedcentral.com/articles/supplements/volume-21-supplement-1.

## References

[1] Ji Z, Jiang X, Wang S, Xiong Li, Ohno-Macha L (2014). Differentially private distributed logistic regression using private and public data. BMC Med Genomics.

[2] Bao W, Huang DS, Chen YH (2020). MSIT: Malonylation Sites Identification Tree. Curr Bioinform.

[3] Bao W, Yang B, Huang DS, Wang D, Liu Q, Chen YH, Bao W (2019). IMKPse: identification of protein malonylation sites by the key features into general PseAAC. IEEE Access.

[4] Ji Z, Zhao W, Lin H, Zhou X (2019). Systematically understanding the immunity leading to CRPC progression. PLoS Comput Biol.

[5] Liu C, Chyr J, Zhao W, Xu W, Ji Z, Tan H, Soto C, Zhou X (2017). Genome-wide association and mechanistic studies indicate that immune response contributes to Alzheimer's disease development. Front Genet.

[6] Shao H, Peng T, Ji Z, Su J, Zhou X (2014). Systematically studying kinase inhibitor induced signaling network signatures by integrating both therapeutic and side effects. PLoS ONE.

[7] Wang M, Ji Z, Kim H, Wang S (2018). Selecting optimal subset to release under differentially private M-estimators from hybrid datasets. IEEE Trans Knowl Data Eng.

[8] Suthaharan S. Characterization of differentially private logistic regression. In: The ACMSE 2018 conference. ACM. 2018. p. 1–8.

[9] Meng X, Zhang X (2015). Big data privacy management. J Comput Res Dev.

[10] Xu L, Jiang C, Wang J, Yuan J, Ren Y (2014). Information security in big data: privacy and data mining. IEEE Access.

[11] Mehmood A, Natgunanathan I, Xiang Y, Hua G, Guo S (2016). Protection of big data privacy. IEEE Access.

[12] Cormode G, Srivastava D, Yu T, Zhang Q (2010). Anonymizing bipartite graph data using safe groupings. VLDB J.

[13] Zhang J, Cormode G, Procopiuc CM, Strivastava D, Xiao X. Private release of graph statistics using ladder functions. In: Proceedings of the 2015 ACM SIGMOD international conference on management of data. ACM. 2015. p. 731–45.

[14] Bhagat S, Cormode G, Krishnamurthy B, Strivastava D (2009). Class-based graph anonymization for social network data. Proc VLDB Endow.

[15] Palanisamy B, Liu L, Zhou Y, Wang Q (2018). Privacy-preserving publishing of multilevel utility-controlled graph datasets. ACM Trans Internet Technol.

[16] Campan A, Traian M. A clustering approach for data and structural anonymity in social networks. In: Privacy, security, and trust in KDD Workshop (PinKDD). 2008. p. 33–54.

[17] Fu H, Zhang A, Xie X (2015). Effective social graph deanonymization based on graph structure and descriptive information. ACM Trans Intell Syst Technol.

[18] Casas-Roma J, Herrera-Joancomartí J, Torra V (2017). A survey of graph-modification techniques for privacy-preserving on networks. Artif Intell Rev.

[19] Zheleva E, Getoor L (2014). Preserving the privacy of sensitive relationships in graph data. Int J Comput Trends Technol.

[20] Aggarwal CC, Li Y, Yu PS. On the hardness of graph anonymization. In: 2011 IEEE 11th international conference on data mining*.* Vancouver, BC. 2011. p. 1002–7.

[21] Horawalavithana S, Gandy C, Flores JA, Skvoretz J, Iamnitchi A, Aiello L, Cherifi C, Cherifi H, Lambiotte R, Lió P, Rocha L (2018). Diversity, homophily and the risk of node re-identification in labeled social graphs. Complex networks and their applications VII. COMPLEX NETWORKS 2018. Studies in computational intelligence.

[22] Karwa V, Slavković A B, Krivitsky P. Differentially private exponential random graphs. In: Privacy in statistical databases. Springer. 2015. p. 143–55.

[23] Sala A, Zhao X, Wilson C, Zheng H and Zhao B Y: Sharing graphs using differentially private graph models. *Proceedings of the 2011 ACM SIGCOMM conference on Internet measurement conference*. ACM, 2011: 81–98.

[24] Medforth N, Wang K. Privacy risk in graph stream publishing for social network data. In: 2011 IEEE 11th international conference on data mining. IEEE. 2011. p. 437–46.

[25] Rossi L, Musolesi M, Torsello A. On the k-anonymization of time-varying and multi-layer social graphs. In: Proceedings of the international AAAI conference on web and social media. 2015. https://ojs.aaai.org/index.php/ICWSM/article/view/14605.

[26] Zhou B, Pei J (2011). The k-anonymity and l-diversity approaches for privacy preservation in social networks against neighborhood attacks. Knowl Inf Syst.

[27] Campan A, Truta TM (2008). Data and structural k-anonymity in social networks. Lect Notes Comput Sci.

[28] Fung BCM, Wang K, Chen R, Yu PS (2010). Privacy-preserving data publishing: a survey of recent developments. ACM Comput Surv.

[29] Office for Civil Rights. HHS: standards for privacy of individually identifiable health information. Final rule, Fed Regist. 2012. http://www.hhs.gov/ocr/privacy/hipaa/administrative/privacyrule/adminsimpregtext.pdf. 12180470

[30] Liu K, Terzi E. Towards identity anonymization on graphs. In: Proceedings of the 2008 ACM SIGMOD international conference on Management of data. ACM. 2008. p. 93–106.

[31] Cheng J, Fu AW, Liu J. K-isomorphism: privacy preserving network publication against structural attacks. In: Proceedings of the 2010 ACM SIGMOD international conference on management of data. ACM, 2010. p. 459–70.

[32] Hay M, Miklau G, Jensen D, Towsley D, Weis P (2010). Resisting structural re-identification in anonymized social networks. VLDB J.

[33] Liu P, Bai Y, Wang L, Li X (2017). Partial k-anonymity for privacy-preserving social network data publishing. Int J Softw Eng Knowl Eng.

[34] Byun JW, Kamra A, Bertino E, Li N. Efficient k-anonymization using clustering techniques. In: International conference on database systems for advanced applications*.* Berlin: Springer. 2007. p. 188–20.

